# Insights on the enigmatic millipede order Siphoniulida (Myriapoda, Diplopoda): a new species bearing ozopores and its phylogenetic implications

**DOI:** 10.7717/peerj.20594

**Published:** 2026-01-22

**Authors:** Ernesto Recuero, E. Karen López-Estrada, Curt W. Harden

**Affiliations:** 1 Museo Nacional de Ciencias Naturales-CSIC, Madrid, Spain; 2 School of Life and Environmental Sciences, University of Sydney, Sydney, NSW, Australia; 3 Unidad de Síntesis en Sistemática y Evolución, Instituto de Biología, Universidad Nacional Autónoma de México, Ciudad de México, Mexico; 4 Plant Industries Division, West Virginia Department of Agriculture, Charleston, WV, United States of America; 5 Department of Plant & Environmental Sciences, Clemson University, Clemson, SC, United States of America

**Keywords:** Taxonomy, Phylogeny, Morphology, Montane cloud forest, Cytochrome C Oxidase I, Helminthomorpha, Dark taxa

## Abstract

The millipede order Siphoniulida is one of the most enigmatic and rare groups within Diplopoda, with fewer than 10 complete specimens known from two extant species and two amber fossils. This study presents the discovery of a new species, *Siphoniulus porosus* sp. nov., from a tropical montane cloud forest in Veracruz, Mexico, representing the highest elevation record for the order in the New World. We obtained the first molecular data for the order, a DNA barcode sequence of the Cytochrome C Oxidase I (COI). Detailed morphological analysis using scanning electron microscopy (SEM) showed that, unlike previously described species, *Siphoniulus porosus* sp. nov. exhibits ozopores, challenging the current understanding that Siphoniulida lack these structures. Phylogenetic analyses using both Maximum Parsimony and Bayesian methods were conducted, including a reassessment of existing morphological data considering the presence of ozopores in Siphoniulida as the ancestral state for this character. The results suggest a phylogentic position within the subterclass Eugnatha, though relationships in this group are not resolved. This discovery indicates a potentially greater diversity of Siphoniulida in the Neotropical Region and highlights the need for further exploration of montane cloud forests to discover additional species.

## Introduction

The millipede order Siphoniulida is probably among the most enigmatic groups of animals on the Planet and, undoubtedly, the rarest of the 16 extant orders of the Diplopoda. Siphoniulidan millipedes are very rarely found, with fewer than 10 complete specimens reported in the literature corresponding to two extant species (Pocock, 1894; Hoffman, 1979; Sierwald et al., 2003), and two amber fossils representing two Cretaceous species (Liu, Rühr & Wesener, 2017). Moreover, they exhibit an unusual morphology, with traits typical of disparate major groups: reduced mouthparts as in the subclass Colobognatha (but see Sierwald et al., 2003), completely fused rings as in the superorder Juliformia, and presence of spinnerets typically found in the superorders Merocheta and Nematophora (Hoffman, 1979; Sierwald et al., 2003; Liu, Rühr & Wesener, 2017). Given the scarcity of specimens and the unusual morphology, the systematic position of Siphoniulida remains unsolved, currently classified as Helminthomorpha *incertae sedis* (Enghoff, 1984; Enghoff et al., 2015).

The group was first introduced by Pocock, who described *Siphoniulus albus* Pocock, 1894 based on a female specimen collected in Sumatra (Pocock, 1894). Its morphological peculiarities did not escape Pocock, and he created a whole new family, while Cook (1895) considered it as a different suborder within Diplocheta and indicated that it would probably deserve its own order. This species has never been collected since then, and nothing more was learned about these organisms for more than 80 years, until *Siphoniulus neotropicus* (Hoffman, 1979) was discovered and described from the other side of the World, in Guatemala, and the order Siphoniulida was formally established (Hoffman, 1979). Hoffman’s species was also based on just two female, likely immature specimens, and a second visit to the same locality in Tikal yielded no new material, highlighting the rarity of these animals (Hoffman, 1979).

Several years later, a small series of *Siphoniulus* aff. *neotropicus* was found at the Field Museum in leaf litter bulk samples collected in the 1980s from three lowland localities in Chiapas and Veracruz, Mexico (Sierwald et al., 2003). A detailed morphological study, including imaging with scanning electron microscopy (SEM) and the first description of male gonopods, allowed a new attempt to infer its phylogenetic position within Diplopoda. Nevertheless, these analyses provided no resolution, and the inclusion of Siphoniulida in the morphological phylogenetic analyses resulted in a largely polytomic Helmintomorpha (Sierwald et al., 2003).

Finally, the last contribution to our knowledge of the order Siphoniulida were two more species, but this time found in Cretaceous Burmese amber, ca. 100 My old, recently described using micro-computed tomography (mCT) technology, and placed in the same family and genus as the extant species, *Siphoniulus muelleri* Liu, Rühr and Wesener, 2017 and *S*. *preciosus* Liu, Rühr and Wesener, 2017 (Liu, Rühr & Wesener, 2017).

Although in the original description of *Siphoniulus albus* it was stated that it has conspicuous pores placed laterally in the middle of the rings (Pocock, 1894), subsequent examination of the holotype (Sierwald et al., 2003) and of specimens of *S*. *neotropicus* (Hoffman, 1979; Sierwald et al., 2003) found no trace of ozopores, a character shared with some other Helmintomorpha groups like Chordeumatida, and some Polydesmida groups where ozopores are secondarily absent (Enghoff et al., 2015). The absence of ozopores in previously studied Siphoniulida is a character state that departs from all other millipede groups with completely fused body rings within the superorder Juliformia, which always present a pair of ozopores opening laterally on most of the body rings (Enghoff et al., 2015).

Like Pocock and Hoffman before us, we find ourselves with regrettably limited material, a single female specimen collected from tropical montane cloud forest in Veracruz, Mexico, the highest altitude occurrence of Siphoniulida in the New World. This new specimen presents unique traits within the order, particularly the presence of ozopores, and we are proposing a new species within the genus *Siphoniulus* Pocok, 1894. The specimen is illustrated with SEM images and accompanied by an associated Cytochrome C Oxidase I (COI) barcode.

## Materials & Methods

**Figure 1:**
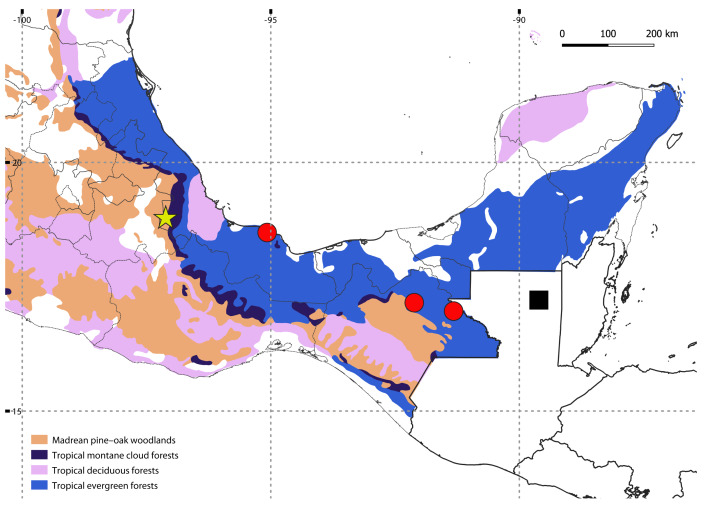
Map showing the known distribution of neotropical Siphoniulida, and associated forest ecosystems in Mexico; *Siphoniulus porosus* sp. nov (yellow star), *S*. *neotropicus* (black square), *S*. aff. *neotropicus* (red circles). Map Source Credit: QGIS v.3.30 (http://qgis.org/) using a publicly available layer of potential vegetation of Mexico (Rzedowski, 1990), obtained from CONABIO’s Geoportal (dataset ID: vpr4mgw, available at: http://geoportal.conabio.gob.mx/metadatos/doc/html/vpr4mgw.html), CC BY-NC 2.5 MX.

A single specimen was found by manually searching in the leaf litter of a tropical montane cloud forest in Orizaba, Mexico, at 1,619 m above sea level (a.s.l.) (Fig. 1); after finding this specimen, the search was intensified and continued for two days more in the same area, but with no success. The specimen was initially preserved in 70% ethanol, and transferred to 100% ethanol after a few months. Collecting permits were issued by Secretaría de Medio Ambiente y Recursos Naturales (SEMARNAT), Mexico (SGPA/DGVS/ 04386/15). The holotype is deposited at the Colección Nacional de Arácnidos (CNAC), Instituto de Biología, UNAM, México. The map showing localities was generated using QGIS v.3.30 (http://qgis.org/), with a layer of potential vegetation of Mexico (Rzedowski, 1990).

The general habitus of the preserved specimen was illustrated by focus stacking 25 pictures taken with a Visionary Digital Passport system equipped with a Nikon EOS 6D camera with a Canon MP-E 65 mm macro lens and a Tamron AF 1.4x teleconverter. Picture stacking was performed with Helicon Focus software v.8.1.1 (HeliconSoft, Ukraine). Morphological examination was performed using an Olympus SZX7 stereomicroscope, and a Hitachi S3400N variable pressure scanning electron microscope (SEM), without metal coating, at the Electron Microscopy Facility, Clemson University, and a Thermo Scientific Apreo 2 field emission scanning electron microscope, also without metal coating, at the Museo Nacional de Ciencias Naturales-CSIC (MNCN).

We reanalyzed the morphological matrix built by Blanke & Wesener (2014), reconsidering the presence of ozopores and the subdivided tarsi as ancestral characters of Siphoniulida (File S1). We ran a Maximum Parsimony (MP) analysis in *PAUP (Swofford, 2003), using Pauropoda as the outgroup and with all 34 characters equally weighted. The analysis was performed using a heuristic search, retaining optimal trees only. Starting trees for branch swapping were obtained by stepwise addition (swapping on best only), and random stepwise addition (1,000 replicates, seed = 1,894), with TBR as the swapping algorithm (reconnection limit = 8). We performed 1,000 bootstrap replicates to assess node support, and constructed a 50% majority-rule consensus tree to visualize phylogenetic relationships. Also, to test the potential effect of character weighting, we performed a Maximum Parsimony analysis in TNT v1.6 (Goloboff & Morales, 2023) using implied weighting (Goloboff, 1993) with *k* = 3 and the same parameters and bootstrap replicates as indicated before. Morphological matrix was also analyzed under a Bayesian framework in MrBayes (Ronquist et al., 2012). For this analysis we partitioned the characters according to their number of states. Recently, it has been demonstrated that if the state space is wrongly assumed to be too large or too small, then branch lengths can be underestimated or overestimated, respectively (Khakurel et al., 2024). The morphological matrix was analyzed using the Mk model (Lewis, 2001) correcting for ascertainment bias (coding = informative) and with equal rates of transition. Analysis consisted of two simultaneous chains of 100,000,000 generations each sampling every 10,000 generations. Convergence and mixing among chains were evaluated by checking the average standard deviation of split frequencies (<0.01) and the effective sample size values for every parameter (>200). To calculate the posterior probability of nodes, we constructed a 50% majority rule consensus tree from the posterior sample after removing the first 25% of the samples as burn-in. Percentage of times a clade occurred among the sampled trees was interpreted as its posterior probability.

DNA sequencing of a 658 bp barcoding fragment of the Cytochrome c oxidase subunit I (COI) gene was performed as explained in Recuero & Rodríguez-Flores (2020), using the universal primers LCO-1490 and HCO-2198 (Folmer et al., 1994), with an annealing temperature of 45 °C in the PCR reaction. The sequence is deposited in GenBank under accession number PX250253 (available at https://www.ncbi.nlm.nih.gov/genbank/) (File S2). The forward and reverse sequences were assembled and revised using Sequencher v.5.4.1 (Gene Codes Corporation), and the final sequence was translated to amino acids to discard the presence of stop codons typical of mtDNA pseudogenes. The nucleotide and amino acid sequences were blasted in GenBank (https://blast.ncbi.nlm.nih.gov/Blast.cgi, accessed on May 21st 2024) against the Diplopoda database to observe patterns of similarity with other orders.

The electronic version of this article in Portable Document Format (PDF) will represent a published work according to the International Commission on Zoological Nomenclature (ICZN), and hence the new names contained in the electronic version are effectively published under that Code from the electronic edition alone. This published work and the nomenclatural acts it contains have been registered in ZooBank, the online registration system for the ICZN. The ZooBank LSIDs (Life Science Identifiers) can be resolved and the associated information viewed through any standard web browser by appending the LSID to the prefix http://zoobank.org/. The LSID for this publication is: urn:lsid:zoobank.org:pub:141770A4-4BED-4FED-8D2E-1F11460D8D3C. The online version of this work is archived and available from the following digital repositories: PeerJ, PubMed Central SCIE and CLOCKSS.

## Results

**Figure 2:**
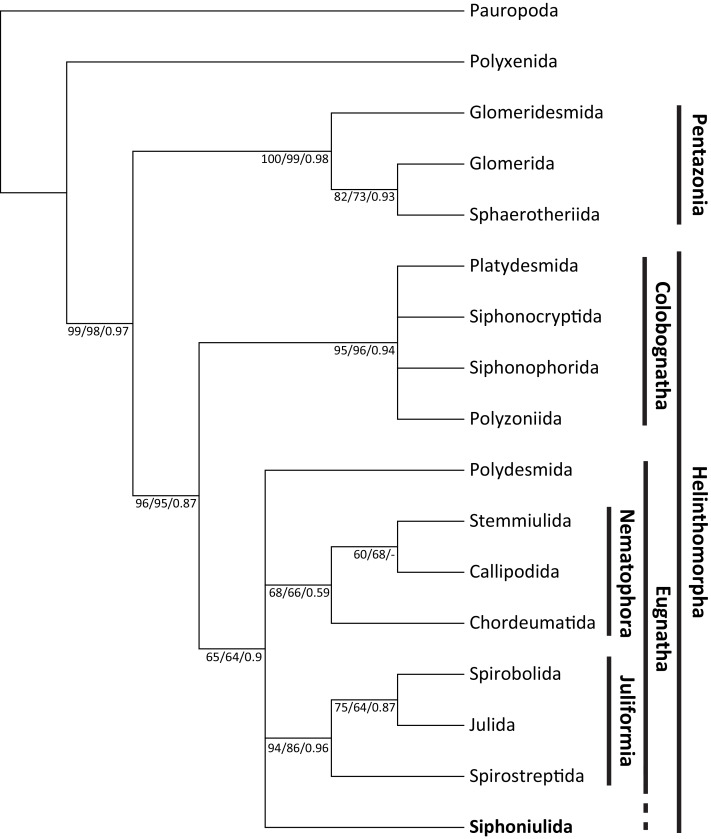
Maximum parsimony tree showing obtained phylogenetic relationships among Diplopoda orders. Maximum parsimony 50% majority-rule consensus tree of 1,000 bootstrap replicates, showing phylogenetic relationships among Diplopoda orders. Numbers above branches indicate unweighted MP bootstrap values/weighted MP bootstrap values/Bayesian posterior probabilities.

The morphological matrix presented 55 parsimony informative characters. The unweighted MP phylogenetic analysis yielded 28 equally most parsimonious trees, in which Siphoniulida was placed as the sister to Juliformia (Spirostreptida, Spirobolida, Julida) in a 75% of cases. However, this relationship with Juliformia is not supported by bootstrap results (also when using implied weighting), nor by Bayesian analysis, and we observe a polytomic clade including Siphoniulida and all Eugnatha orders, with relationships between Siphoniulida, Polydesmida, Nematophora and Juliformia not resolved (Fig. 2). Support for clades Pentazonia, Helminthomorpha, Colobognatha, and Juliformia is high in both analyses. This support is lower for the clades Eugnatha (although relatively strong in the Bayesian tree) and Nematophora (Fig. 2).

The barcoding COI fragment showed no stop codons, indicating that it does not correspond to a degenerated nuclear pseudogene. Blasting the sequence in GenBank against the Diplopoda database yielded similar matches with members of different orders, as expected due to saturation among distantly related taxa. The highest percentual identities (80–81%) were observed with members of Polydesmida, while values of 75–79% were observed also with members of Spirostreptida, Spirobolida, Julida, Polydesmida, Chordeumatida, and even Sphaerotheriida and Polyxenida. The matches with lowest E-values (the number of expected hits of similar quality (score) that could be found just by chance) were with members of Juliformia. Similarly, when blasting the amino acid sequence, the best matches in terms of percent identity and E-values were with members of Juliformia.

### Taxonomy

**Table:** 

Class Diplopoda de Blainville in Gervais, 1844
Subclass Chilognatha Latreille, 1802/1803
Infraclass Helminthomorpha Pocock, 1887
Subterclass Eugnatha Attems, 1898
Order Siphoniulida Pocock, 1894
Family Siphoniulidae Pocock, 1894
Genus *Siphoniulus* Pocock, 1894

**Table:** 

**Figure 3:**
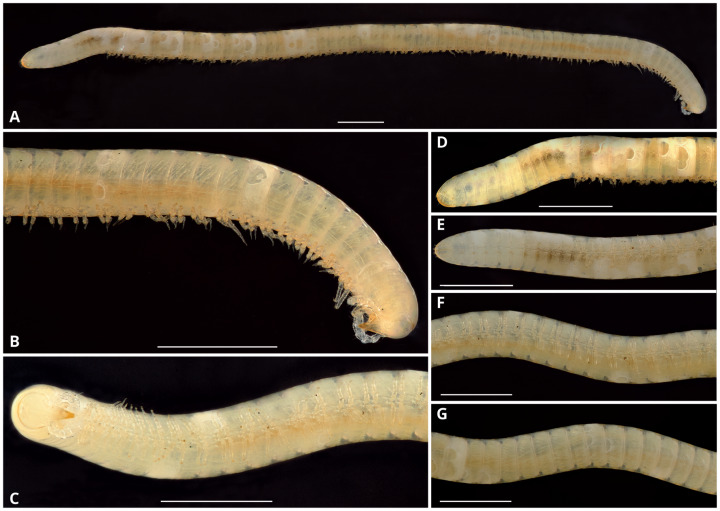
*Siphoniulus porosus* sp. nov., general habitus of female holotype (CNAC). (A) Whole specimen, lateral view. (B) Anterior part of body, lateral view. (C) Anterior part of body, ventral view. (D) Posterior part of body, lateral view. (E) Posterior part of body, ventral view. (F) Midbody segments, ventral view. (G) Midbody rings, dorsal view. Scale bars = one mm.

**Figure 4:**
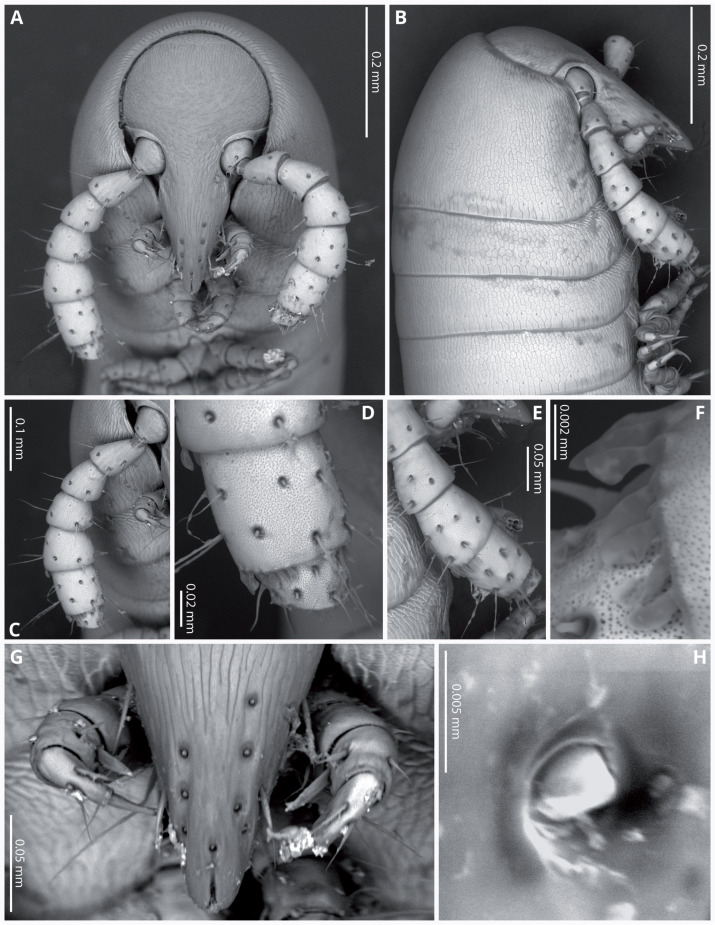
*Siphoniulus porosus* sp. nov., SEM images of female holotype (CNAC). (A) Head, antennae and anterior rings, anterior-ventral view. (B) Head, antenna and anterior rings, lateral view. (C) Right antenna, frontal view. (D) Antennomeres 5-7, frontal view. (E) Right antenna, lateral view. (F) Laminar sensilla on antennomere 6. (G) Clypeal region, showing button-like sensilla, and first leg pair. (H) Close-up of a button-like sensillum on clypeal region.

**Figure 5:**
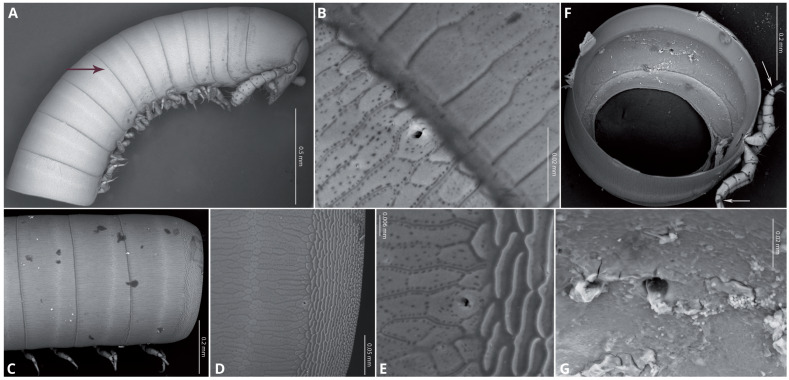
*Siphoniulus porosus* sp. nov., SEM images of female holotype (CNAC). (A) anterior part of body, lateral view (black arrow indicates position of first ozopores); (B) close-up of ozopore on ring 8; (C) midbody rings, lateral view; (D) anterior part of ring 16 on lateral view, showing prozonite, metazonite and ozopores; (E) close-up of ozopore on ring 16; (F) Internal view of a midbody ring wit soft tissues digested (white arrows indicate the suture-like structure in the leg tarsi); (G) close-up of ozopore on midbody ring, internal view.

**Figure 6:**
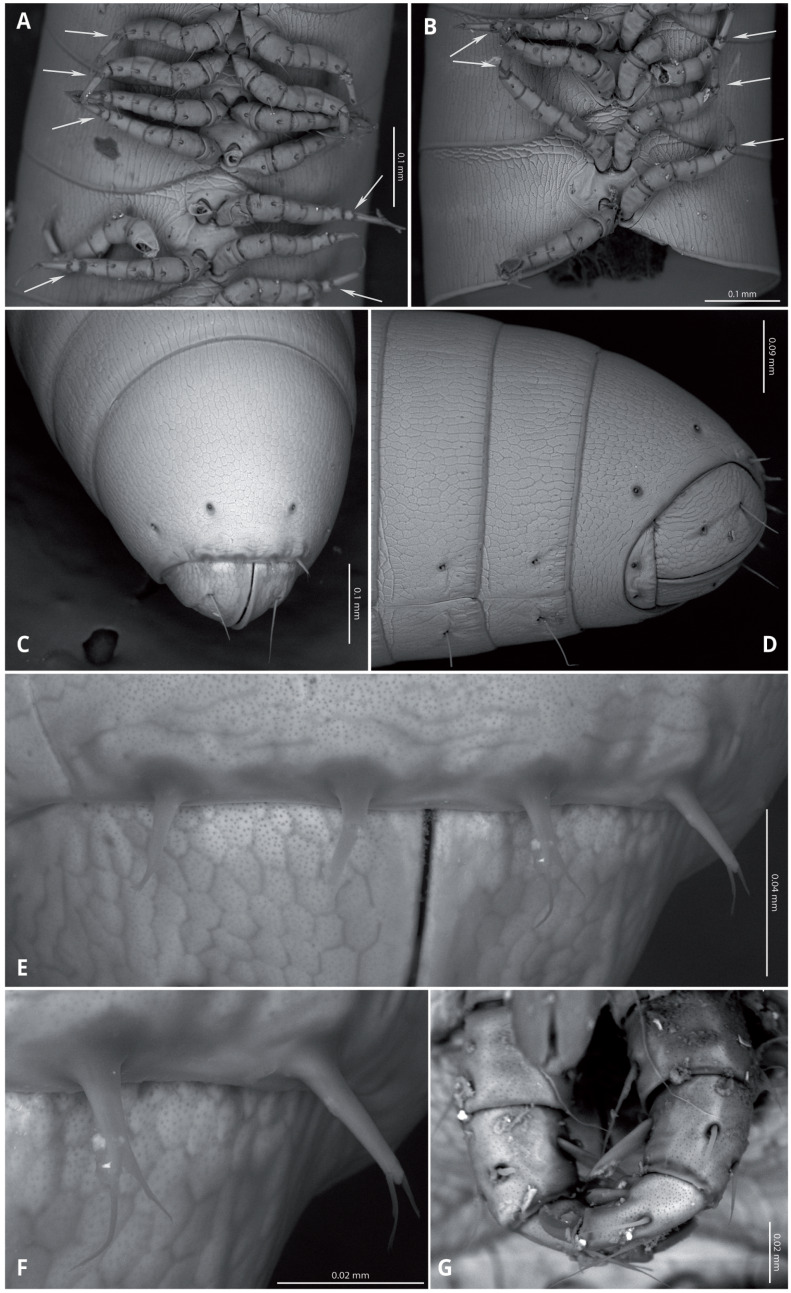
*Siphoniulus porosus* sp. nov., SEM images of female holotype (CNAC). (A, B) ventral view of midbody rings showing walking legs and sternites (white arrows indicate the suture-like structure in the leg tarsi); (C) terminal rings, dorsal view; (D) terminal rings, ventrolateral view; (E, F) close-up of spinnerets; (G) second leg pair.

***Siphoniulus porosus*** sp. nov.
urn:lsid:zoobank.org:act:F510ECDD-1B87-45EE-87DC-27E3D91A68E7
Figs. 3–6

**Material examined**

**Holotype**

Mexico •♀; Veracruz, Orizaba; 18°51’N 97°07’W; 1619 m a.s.l.; 23 Jan. 2016; E.K. López-Estrada, E. Recuero leg.; CNAC.

**Diagnosis**: This species is distinguished from other extant *Siphoniulus* by the presence of ozopores and the tarsi subdivided by a suture. It differs also from *Siphoniulus neotropicus sensu stricto* in having a very short and broad antennomere 7, also with short apical cones, longer and slenderer in the latter species (but not in *S*. aff. *neotropicus* of Sierwald et al., 2003).

**Etymology**: From the Latin *porosus* (with pores) referring to the presence of ozopores.

**Description**: Total length of the holotype, 14.7 mm, maximum diameter, 0.6 mm. 74 rings including collum, 63 podous rings, 9 apodous rings and telson. Unpigmented, the whole specimen showing an off-white coloration (Fig. 3).

Head (Figs. 4A, 4B) deflexed, pyriform in anterior view, without eyes; vertex without sulcus; frons long, tapering and ending in a narrow, rounded labrum with a deep median cleft; vertex without setae, clypeal region bearing eight sub-aligned button-like sensilla (Figs. 4G, 4H); no trace of Tömösvary organ; mouth parts not examined. Antennae (Figs. 4C–4F) set frontolaterally on head, about as long as head and moderately robust on their distal half; interbasal space 1.7 times the length of 1st article; seven antennomeres, with relative lengths as 2>6>5>4=3>1>7; all antennomeres with sparse medium to long setae; 5th and 6th antennomeres with series of five and 10 laminar sensilla respectively, located posterolaterally; 7th very short and broad. bearing the apical sensory apparatus with 4 acute antennal cones, set closely together.

Collum long, dorsally longer than tergites two and three combined (Figs. 3B, 4B), with anterior margin covering the base of head and folding ventrally towards the base of the first pair of legs (Fig. 4A).

Body very elongate and slender (Fig. 3A). Rings with a narrow prozonite, with scale-like surface, and a broad metazonite with coriaceous texture (Fig. 5); ozopores present from ring 8 (Figs. 5A, 5C) very small, located laterally right behind the prozonite, and are often almost covered by the preceding ring (Figs. 5A–5E); internally, the ozopore opening is flush with the exoskeleton surface (Figs. 5F, 5G); no median dorsal suture; pleural and tergal sclerites fused with no sutures, sternites fused with pleurotergum with suture lines still evident (Figs. 6A, 6B). Anterior sternite narrower than posterior one. Stigmata present on both sterna, slit-like, but the posterior one showing a small rounded pit (Figs. 6A, 6B). Rings 2 and 3 not completely closed, the first two leg-pairs carried by free sterna; following rings to ring 64 with two pairs of legs, last nine trunk rings legless, with a pair of paramedian, ventral, long setae (Fig. 6D). Epiproct (Figs. 6C, 6D) not produced, carrying two lateral pairs of setae and four short, broad spinnerets in the caudal margin (Figs. 6E, 6F); paraprocts convex, coriaceous, with two large setae each; hypoproct short, with straight caudal margin and two paramedian setae (Fig. 6D).

Walking legs (Figs. 6A, 6B) short and relatively robust, not extending beyond the sides of the body, each composed of six short podomeres. Coxae longer and distally expanded; femur, postfemur and tibia subequal in length and shape; tarsus slenderer and about as long as postfemur and tibia combined, with a suture-like structure near the base that divides this podomere in two sections (Figs. 4F, 5A, 5B). However, no articulation associated with this suture was observed under an optical microscope (up to 40 × magnification), so it may represent a transverse band of less sclerotized cuticle. Tarsal claw about as long as the tarsus, with basal accessory unguis; most podomeres with two ventrolateral long setae. Legs 1 and 2 with five podomeres, shorter and thicker than walking legs; tibia with a large ventral, spatulate seta; tarsus with two, long but regular-shaped setae (Fig. 6G).

**Habitat**: The new species was found living in leaf litter in a remnant of tropical montane cloud forests. Litter was humid and over 25 cm deep, with the deeper layers very fragmented and with numerous roots. *Siphoniulus porosus* inhabits a different ecosystem than *S*. *neotropicus*, a species known only from tropical evergreen forests at low altitudes (Fig. 1).

**Remarks**: Even in the absence of a male specimen, *Siphoniulus porosus* sp. nov. is unique among the known extant members of the order in having ozopores on the midbody podous rings from ring 8. These ozopores are minute and hardly distinguishable unless using very high magnification, and it is possible that they can be partially hidden under the previous ring. However, Hoffman (1979) verified the absence of ozopores in *Siphoniulus neotropicus* by clearing in glycerin and examining the specimens at 430X magnification, while Sierwald et al. (2003) did not observe them in specimens tentatively assigned to *S*. aff. *neotropicus*, even in SEM pictures, so it is unlikely that they have been overlooked. Even if Pocock (1894) mentions the presence of conspicuous ozopores in *Siphoniulus albus*, both Hoffman and Sierwald were able to examine the holotype of *Siphoniulus albus*, finding no trace of ozopores (Hoffman, 1979; Sierwald et al., 2003), even at 100x magnification. This may suggest that this species have also extremely small ozopores, hardly detectable at lower magnifications, but that could be detected in the freshly fixed specimen by the color of associated glands, as frequently happens in other millipede species. In the case of *Siphoniulus porosus* sp. nov., we observed no trace of color indicating the presence of ozadenes even when the specimen was alive. In the Cretaceous species descriptions, there is no mention of ozopores (Liu, Rühr & Wesener, 2017), but if present they are probably unappreciable through the amber where they are enclosed.

*Siphoniulus porosus* sp. nov. also presents tarsi divided by a suture-like structure that is not observed or mentioned in the other species of the genus, and a particularly short and broad antennomere 7, differing from that of *S. neotropicus*, clearly longer and slenderer.

Regarding the spinnerets, they differ from those observed in *S*. aff. *neotropicus* (Sierwald et al., 2003). In the latter, they appear as basally swollen, with the distal part tapering into an acute point; In *S*. *porosus* sp. nov, they are only slightly broader at the base, and the tip, if not broken, is clearly bifurcated.

Considering the distinctive features of the new species, it is likely to represent at least a new genus within Siphoniulida. However, given the scarcity of information on all known species, we refrain from making this decision until a full systematic review of the group can be performed.

## Discussion

The discovery of a new member of Siphoniulida in tropical forests of eastern Mexico is not so surprising, as the group had already been found in a few localities not so distant from the type locality of *Siphoniulus porosus* sp. nov. (Fig. 1). The closest known locality where the order has been reported is a little over 200 km to the southeast, in the Estación de Biología Tropical Los Tuxtlas (Sierwald et al., 2003). However, all previous neotropical records of the order were made in low elevation tropical evergreen forests, at elevations between 20 and 700 m a.s.l. (Hoffman, 1979; Sierwald et al., 2003), while the new species has been collected at a much higher elevation and in a different mountainous tropical biome, the montane cloud forest. This particular ecosystem is restricted at mid to upper elevations, where clouds and mists maintain a constant high humidity, and is characterized by its outstanding biodiversity, including a high number of endemic species (Karger et al., 2021). Considering this, it is likely that the diversity of Siphoniulida in Mexico and Central America is much higher than we currently know. Given the sporadic nature of the available observations, and the elongated body and short legs of these animals, it is likely that they have marked fossorial habits, hampering new captures. Exploring the shallow subterranean habitats of these forest could help discover new populations and species, as has happened with other deep soil millipedes (*e.g.*, Gilgado et al., 2015; Marek et al., 2021).

The existence of ozopores in *Siphoniulus porosus* sp. nov. marks this species as unique within the order (Hoffman, 1979; Sierwald et al., 2003, but see Pocock, 1894). Ozopores and associated defense glands (ozadenes) are present in most major groups of Chilognatha, although they may not be homologous in all cases, particularly between Pentazonia and Helminthomorpha (Blanke & Wesener, 2014). In the former, ozopores are absent in Glomeridesmida and Sphaerotheriida, and in Glomerida they are unpaired and opening along the dorsal midline; in the latter, they are paired and opening in the lateral part of podous rings (Eisner et al., 1978). Paired ozopores are present in all orders of Helminthomorpha except Chordeumatida (Eisner et al., 1978). Cases of secondary loss of ozopores are observed also within Polydesmida, where they seem to have completely disappeared in the family Sphaeriodesmidae Humbert & de Saussure, 1869 (Enghoff et al., 2015). Reduction in the number of pores has also been observed in different groups, as in genera and species in the Polydesmida families Pyrgodesmidae, Holistophallidae (reduced to a single pair in the genus *Duoporus* Cook, 1901) and Campodesmidae Cook, 1896, or in the Julida family Mongoliulidae (Hoffman, 1980; Hoffman, 2008; Enghoff et al., 2015; Enghoff, Jensen & Mikhaljova, 2017).

Even if a more detailed examination of additional material is needed, we have found no trace of ozadenes in the examined specimen. This could mean a strong reduction or even loss of them, indicating a trend in the group of secondarily losing their chemical defenses, culminating in the disappearance of ozopores in some species. Given the presence of ozopores in *Siphoniulus porosus* sp. nov., a secondary loss in *S*. *neotropicus* and, perhaps, *S*. *albus*, is the most plausible explanation. In this case, the presence of ozopores would be the ancestral state for the group. If we consider this option and reanalyze the morphological matrix studied by Blanke & Wesener (2014), the resulting phylogenetic hypothesis suggests a closer relationship of Siphoniulida with other Eugnathan orders. A relationship with Juliformia was hypothesized by Hoffman (1979), considering the characters “sterna, pleura, and terga fused into compact rings; shape of the coxal podomere; interruption of the anterior leg series”, and suggesting that absence of ozopores could be considered an apomorphic character state. However, even if most of our most parsimonious trees place Siphoniulida as sister group of the Juliformia, we found no bootstrap support for such a phylogenetic placement. The presence of characters such as spinnerets and segmented tarsi could indicate a closer relationship to the Nematophora, particularly Stemmiulida and Callipodida. In this case, the completely fused ring would be a homoplasy with respect to the Juliformia.

Indeed, while our results indicate that Siphoniulida is clearly a member of the subterclass Eugnatha, its inclusion in morphological phylogenetic analysis of Diplopoda results in several clades with relatively lower support, compared to when it is excluded (Sierwald et al., 2003; Blanke & Wesener, 2014). This is presumably due to its diverse array of morphological traits, as well as the incomplete knowledge of this order. For instance, characters related to its reproductive biology, ontogeny, and chemical defenses are still unavailable. Additional data will be necessary to resolve the relationships of Siphoniulida within Eugnatha.

## Conclusions

*Siphoniulus porosus* sp. nov. represents an outstanding discovery that challenges key morphological assumptions for the group, particularly the presence of ozopores as in most orders of the Diplopoda. For the first time, we present molecular data for the whole order, which will hopefully enable future genetic comparisons within Siphoniulida. The finding of this species in a different forest ecosystem than previously known, points to a potentially greater diversity and wider distribution of the order in the Neotropical region, underscoring the importance of further exploration of montane cloud forests to uncover additional species. Future work should expand the available data to resolve the phylogenetic position of Siphoniulida within Eugnatha.

##  Supplemental Information

10.7717/peerj.20594/supp-1 Supplemental Information 1 Morphological matrix for phylogenetic reconstruction of Diplopoda

10.7717/peerj.20594/supp-2 Supplemental Information 2 COI Barcode for Siphonilus porosus sp. nov

## Additional Information and Declarations

### Field Study Permissions

The following information was supplied relating to field study approvals (i.e., approving body and any reference numbers): Collecting permits were issued by Secretaría de Medio Ambiente y Recursos Naturales (SEMARNAT) (SGPA/DGVS/ 04386/15).

### DNA Deposition

The following information was supplied regarding the deposition of DNA sequences: The dataset is available *via* GenBank accesion number PX250253.

### Data Availability

The following information was supplied regarding data availability: The matrix for phylogenetic reconstruction is available in the Supplementary File. The dataset is available *via* GenBank accesion number PX250253.

### New Species Registration

The following information was supplied regarding the registration of a newly described species: Species name: urn:lsid:zoobank.org:act:F510ECDD-1B87-45EE-87DC-27E3D91A68E7 Publication LSID: urn:lsid:zoobank.org:pub:141770A4-4BED-4FED-8D2E-1F11460D8 D3C.
